# Research hotspots and frontiers of ethnic cultural identity——based on analysis of “web of science” database

**DOI:** 10.3389/fpsyg.2023.1276539

**Published:** 2023-11-15

**Authors:** Lidan Kuang, Xingmei Gao, Bingliang Liu, Jianzhan Wang

**Affiliations:** ^1^Marxism School, China Pharmaceutical University, Nanjing, China; ^2^School of Foreign Languages, China Pharmaceutical University, Nanjing, China; ^3^Business School, Ludong University, Yantai, China

**Keywords:** ethnic cultural identity, CiteSpace, acculturation, cultural adaptation, psychological adaptation

## Abstract

Cultural identity is of great significance to the formation of group consensus and the establishment of cultural self-confidence. In order to understand the history, current situation and trend, and provide theoretical support for future research, this paper makes a quantitative analysis of knowledge map including annual publication volume, trend, distribution of authors and institutions, co-occurrence, clustering and timeline of keywords as well as emergent keywords on the literature concerning ethnic cultural identity published in “Web of Science” database for a period from 2012 to 2022, with CiteSpace software as a tool. The results show an overall upward trend with diversified ethnic and regional characteristics; major institutions including universities of the U.S., the U.K., Australia, China and other countries and regions engage in their research from different disciplines such as psychology, sociology, ethnology and education; the researchers have not formed a core group of authors despite their accumulating number; research hotspots are indicated by keywords such as national identity, identity, ethnic identity and attitude; specifically, keyword clusters fall into three categories: emotional perception, multicultural identity process and ethnic cultural adaptability; researchers probe into various issues at different stages with direct relation to international situations and regional cultures. This study has positive implications for understanding and mastering the current research hotspots and development trends of ethnic cultural identity in the world.

## 1. Introduction

Cultural identity is an individual’s recognition of a group’s attitudes, feelings, degree of belonging (Berry et al., 1989), ideals and values (Schwartz et al., 2006), and other social identities such as class, nationality and race, which is of enormous importance to a culture’s unity, consensus and self-confidence (Villarroya, 2012; Över, 2016; C. Zhang et al., 2019; Zhang et al., 2021; Azada-Palacios, 2022). It is the foundation of cultural self-confidence, national stability and unity for a country to enhance its cultural identity (Schwartz et al., 2006; Li et al., 2015; Waechter, 2015; Yuan et al., 2022). However, numerous studies have shown that it is not easy to construct cultural identity (Minnaert, 2014; Van Der Zwet, 2016; Grajzl et al., 2018; Chitima, 2022). Due to obvious differences between groups and individuals in terms of geographical space, historical and cultural environment, etc., a country has different cultural cognitions, attitudes, ideals, beliefs and even values, profoundly affecting people’s lifestyles, behavior patterns, and emotional expressions, which consequently poses big challenges to form cultural identity and strengthen national unity. With complicated situations and escalated conflicts, cultural identity research has gained increasing worldwide attention from scholars.

Previous studies on ethnic cultural identity have mainly focused on its definition, process of acculturation, changes in psychology and behavior, and perceived discrimination, most of which have been investigated in local situations from one discipline. Few scholars have used bibliometric software to analyze the entire research hotspots and trends. For this reason, based on the literature on cultural identity included in “Web of Science” (WOS) database from 2012 to 2022, this paper uses the bibliometric CiteSpace software (6.2.R3) to conduct a quantitative analysis of trends, author and institution distribution, keyword co-occurrence, clustering and timeline, and emerging keywords, combined with systematic sorting of current affairs politics, cultural policy, cultural adaptation, to analyze the literature concerning cultural identity. This paper aims to explore the culture identity research’s evolution process, grasp its hotspots, and predict its future development trends to provide implications for other scholars.

## 2. Research methodology

CiteSpace software, the abbreviation of Citation Space, is an information visualization software developed by Dr. Chaomei Chen, who is a professor of computer and information science at Drexel University in the United States. Based on Java language and citation analysis theory, it can visualize document data through co-occurrence analysis, co-citation analysis and other methods, so that the relationship between certain items including authors, institutions and keywords can be presented in the form of a scientific knowledge map, which clearly shows the evolution path of a certain subject field CiteSpace. This study uses CiteSpace software (6.2.R3) to analyze the data from the sub-databases of Web of Science (WOS), the comprehensive online literature database in the United States, namely Social Science Citation Index (SSCI) and Science Citation Index Expanded (SCI-Expanded). The selected literature types include English-written research and review papers from January 2012 to December 2022, with the collection and retrieval method of “Topic = cultural identity AND national identity.” A total of 2,824 relevant documents are obtained through retrieval; because some documents are relatively weak, the research field is limited to psychology, social sciences, educational research, sociology, ethnic studies, cultural studies, regional studies, etc. After manually reading and reviewing the titles, abstracts and keywords of the documents, 1,012 valid documents were finally obtained.

## 3. Analysis of research hotspots and frontier

### 3.1. Analysis of publication volume

**Figure 1:**
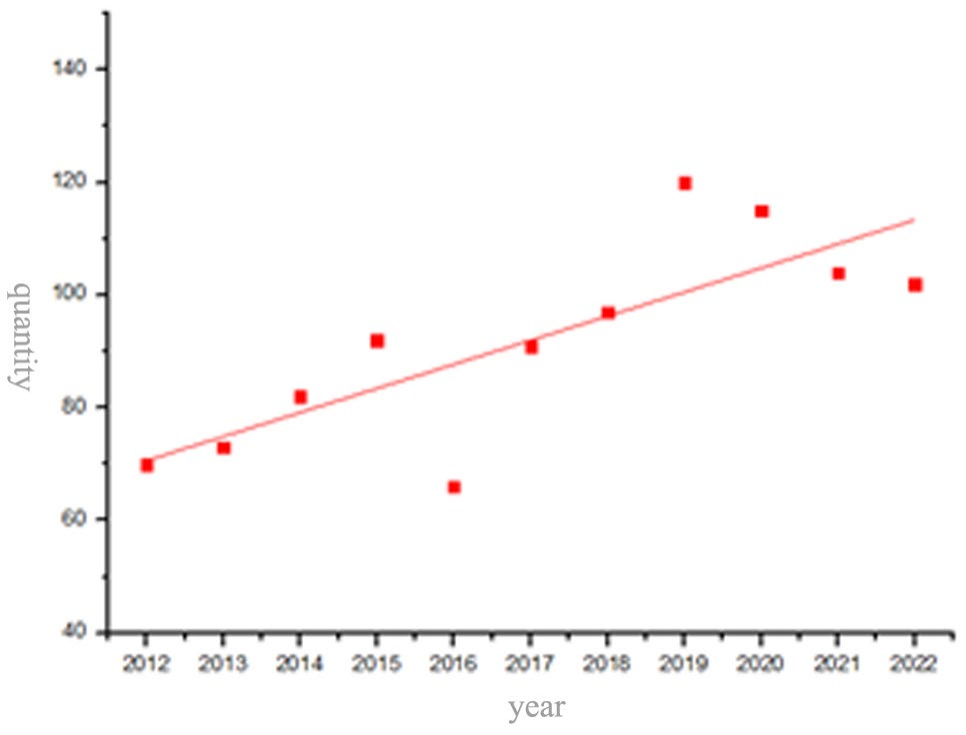
Annual publication volume of research on ethnic cultural identity.

The publication volume is an indicator that effectively reflects the popularity of a specific topic within a certain period of time, and visually shows its development trend. Based on the literature downloaded from WOS database, a yearly distribution map on ethnic cultural identity research is drawn in Figure 1.

Figure 1 shows the general rising trend despite its fluctuations in the publication volume from 2012 to 2022. The number of papers published is 70 in 2012, and reaches a peak of 120 in 2019. Although the number of papers published in other years fluctuates slightly, the overall trend is upward. The data reflect the continuous concern of the international community on the issue of cultural identity. Among these documents, 996 are research papers and 16 are review papers. The researchers, who mainly come from the United States, Britain, Australia, China, Germany and other countries, focus on ethnology and psychology, followed by sociology, pedagogy, cultural studies and other disciplines, with diversified cross-discipline methods comprising mixture-analysis, meta-analysis, structural equation modeling and literature analysis.

The research features apparently differentiated national and regional characteristics, such as immigration issues in the U.S., South Korea, the U.K., Russia and other countries (Ha and Jang, 2015; Canefe, 2018; Grajzl et al., 2018; Grigoryan and Ponizovskiy, 2018), cultural policy issues in Spain, the Netherlands, Iran and other countries (Villarroya, 2012; Minnaert, 2014; Attarzadeh and Seyfodini, 2022), post-colonial contexts (Chitima, 2022), religious beliefs (Younis and Hassan, 2019), cultural tourism (Över, 2016), ethnic identity (Van Der Zwet, 2016; Christophe et al., 2020). Chinese scholars are interested in cultural festivals (Zhang C. X. et al., 2019), bicultural identity integration (Long et al., 2021), national unity education (Luo, 2019), dual identity adaptability (Dai et al., 2018), collective memory and traditional culture (Hou, 2021), cultural heritage (Wu et al., 2022). They appeal to strengthening national unity education, inheriting national culture, and enhancing national cohesion, which demonstrates the current research hotspots and development trends.

### 3.2. Analysis of authors and institutions

#### 3.2.1. Analysis of author cooperation

**Figure 2:**
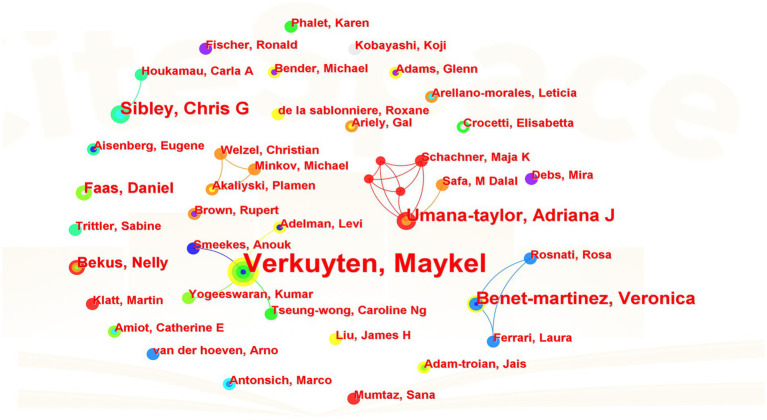
Knowledge map of authors.

In the CiteSpace software, author is set as the node type, and the parameters are Top *N* = 20, Top *N*% = 20.0%, g-index = 25. As shown in Figures 2, a total of 299 author nodes (*N* = 299) are generated, the number of connections between authors is 74 (*E* = 76), and the network co-occurrence density is 0.0017 (*D* = 0.0017). The index shows a low cooperation concentration of authors in spite of their growing number. Among them, authors with a large number of publications and significant cooperative relationships include Maykel Verkuyten, Anouk Smeekes, Kumar Yogeeswaran, Caroline Ng Tseung-Wong, Olivia Spiegler, Levi Adelma, etc., who come from Utrecht University, University of Canterbury, University of Mauritius, University of Oxford, and who focus on cultural belonging, religion and immigration. Among these significant cooperative relationships, scholars make collaborate research within a tiny number of countries, which shows the urgency that scholars strengthen international exchanges and cooperation in this research field.

#### 3.2.2. Analysis of core authors

Tables analyzing the number of publications and research directions of the core authors can better demonstrate the overall trend of the discipline. Here, De Solla Price (1969) formula is used to select core author candidates. The formula is:


$$M_{p}=0.749*\sqrt{N_{\mathrm{pmax}}}$$

In the formula, M_p_ is the minimum number of articles published by core author candidates, and N_pmax_ is the highest number of articles published by authors within the selected literature (Liu et al., 2015). The results show that M_p_ = 2.247 is calculated, taking an integer of 3, indicating that during the period from 2012 to 2022, the number of papers published by the core author candidates is no less than 3.

The number of citations is also an important indicator to measure the quality of a paper, and the citation frequency indirectly indicates the influence and attention of the paper (Li, 2008). The minimum publication volume is calculated according to the Price formula:


$$M_{c}=0.749*\sqrt{N_{\mathrm{cmax}}}$$

M_c_ refers to the minimum number of citations of a single paper, and N_cmax_ refers to the maximum number of citations of a single paper (De Solla Price, 1969).

The statistics show that the maximum number of citations is 251, M_c_ ≈ 11.866, taking an integer of 12, and that those who meet the above two indicators at the same time are the core authors, namely Verkuyten Maykel, Sibley Chris G, Benet-martinez Veronica, Faas Daniel, and Bekus Nelly. These authors mainly come from Utrecht University, University of Auckland, Catalan Institute for Advanced Research, Trinity College, University of Exeter. Among them, Verkuyten Maykel has the largest number of publications with a total of 139 citations. Most of his articles are related to nationality, ethnicity and culture. In one article published in 2020, he points out the enormous importance of developing a sense of identity and common belonging to strengthen community cohesion in diversified multi-culture contexts (Velthuis et al., 2020), which has laid a good foundation for the following research.

According to Price’s law, when the number of published papers by core authors reaches 50% of the total number of published papers, it means that a core group of authors has been formed in this field (Liu et al., 2015). However, the number of papers published by the above-mentioned core authors only takes up 2.3% of the total, significantly lower than this standard. Therefore, it can be seen that the current research has not yet formed a core author group, and that the cooperation is relatively scattered. But at the same time, it also shows that the authors have diversifications without effective cooperation.

#### 3.2.3. Analysis of research institutions and countries

**Figure 3:**
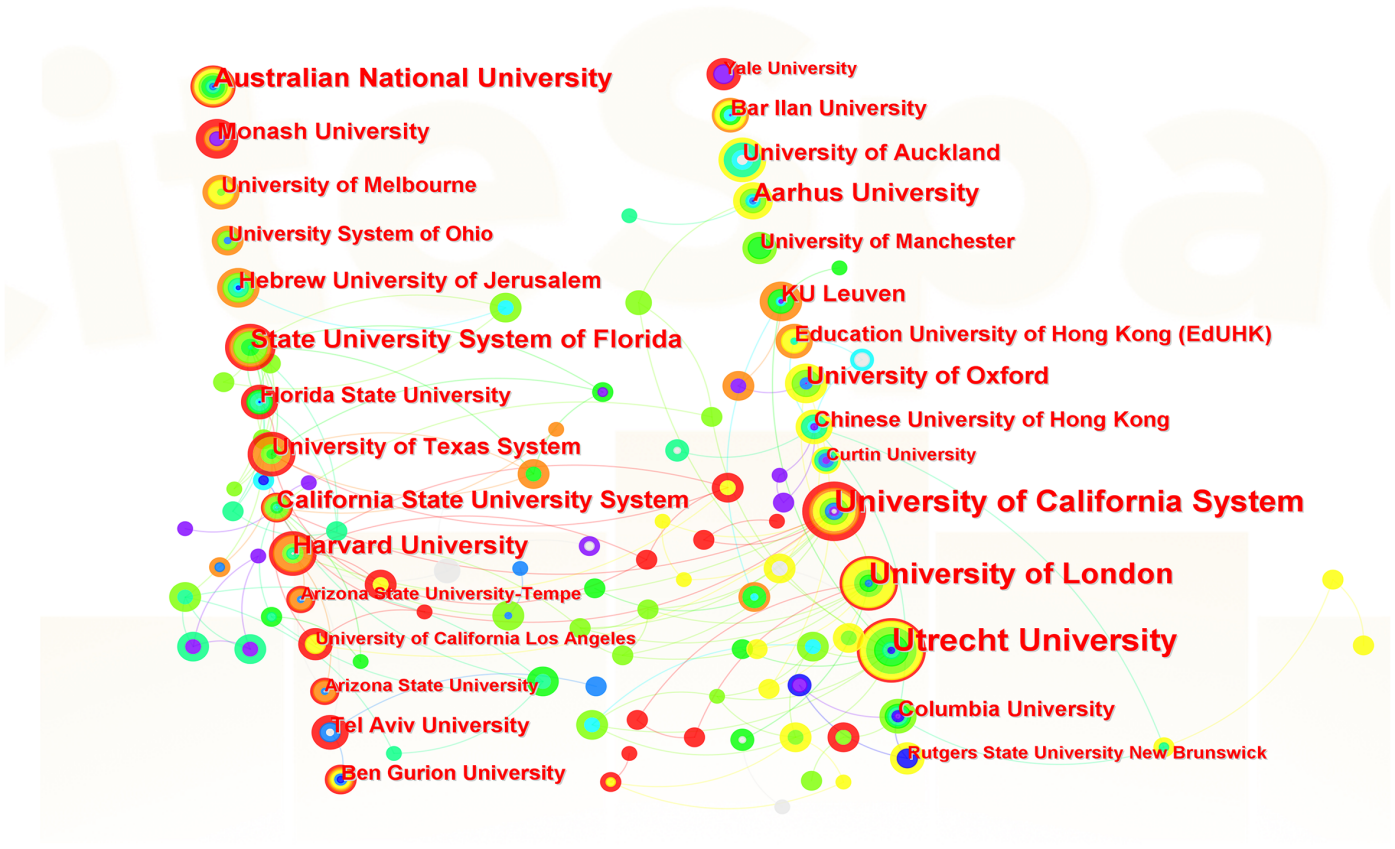
The knowledge map of author’s institutions.

According to the knowledge map of author’s institutions (Figure 3) and the top 21 foreign institutions (Table 1) in terms of the number of published papers, Utrecht University ranks the first with the largest number of 24 papers, followed by California State University with 20 papers and University of London with 18 papers, respectively. The authors mainly come from the universities of the U.S., the U.K., Australia, China, Germany, Israel, the Netherlands, New Zealand, South Korea, Spain, Denmark, Italy, Turkey and Belgium (Table 2). Among them, 276 papers come from the U.S., followed by the U.K. (141), Australia (97), China (85), and Belgium (18). Major concerns include race relations (Neblett et al., 2012; Yogeeswaran et al., 2014; Gökay and Hamourtziadou, 2016; Grajzl et al., 2018; Walton et al., 2018; Huguley et al., 2019), culture conflict (Keddie, 2014; Dupré, 2018; Son, 2019; Tseung-Wong et al., 2019), immigration (Però, 2013; Morrice, 2017; Mole, 2018), social welfare (De La Sablonnière et al., 2020), economic development (Storm, 2018), cultural policy (Villarroya, 2012; Minnaert, 2014), mental health (Howe et al., 2014; Weber et al., 2021), cultural identity (Zhang S. et al., 2019), identity (Chen et al., 2019) and sense of belonging (Zou et al., 2021).

**Table 1:** Foreign institutions with no less than 7 publications on ethnic cultural identity.

No.	Quantity	Neutrality	Time	Institution
1	24	0.1	2012	Utrecht University
2	20	0.08	2012	University of California System
3	18	0.03	2013	University of London
4	13	0	2013	Australian National University
5	12	0.07	2012	Harvard University
6	12	0.03	2017	State University System of Florida
7	11	0	2013	Aarhus University
8	10	0.12	2013	California State University System
9	10	0.01	2013	University of Oxford
10	9	0	2012	University of Auckland
11	9	0.03	2018	University of Texas System
12	8	0	2014	Hebrew University of Jerusalem
13	8	0.02	2013	KU Leuven
14	8	0	2013	Monash University
15	7	0	2013	Bar Ilan University
16	7	0.06	2013	Chinese University of Hong Kong
17	7	0.02	2013	Columbia University
18	7	0	2016	Education University of Hong Kong
19	7	0	2014	Florida State University
20	7	0.01	2012	Tel Aviv University
21	7	0	2019	University of Melbourne

**Table 2:** Country distribution of ethnic cultural identity research (unit: articles).

No.	Country	Quantity
1	USA	276
2	ENGLAND	141
3	AUSTRALIA	97
4	PEOPLES R CHINA	85
5	CANADA	59
6	GERMANY	51
7	ISRAEL	50
8	NETHERLANDS	50
9	NEW ZEALAND	35
10	SOUTH KOREA	25
11	SPAIN	25
12	DENMARK	24
13	ITALY	24
14	TURKEY	23
15	BELGIUM	18

### 3.3. Analysis of research hotspots

#### 3.3.1. Analysis of keyword co-occurrence

**Figure 4:**
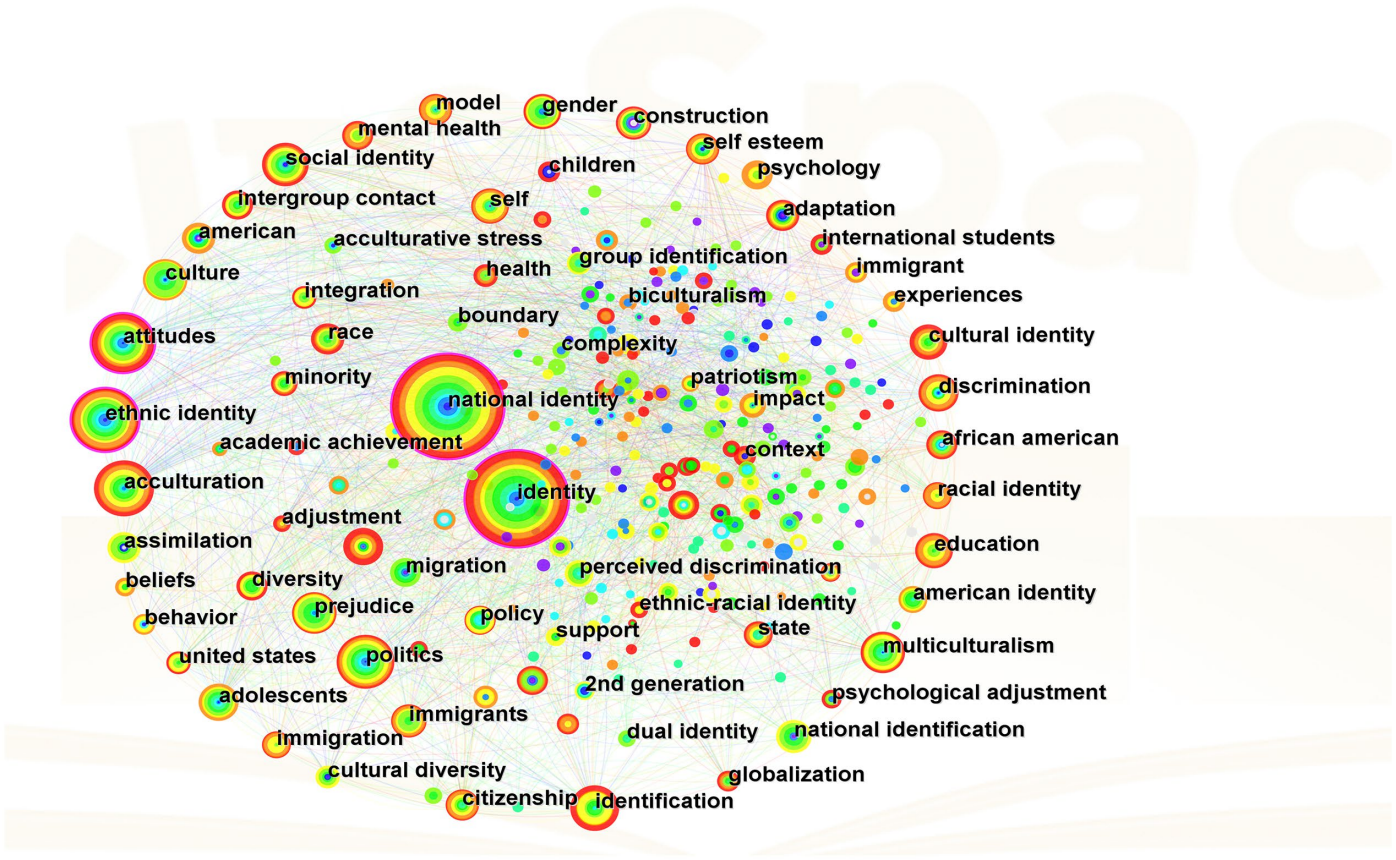
Knowledge map of keyword co-occurrence.

Being highly condensed and generalized, key words can enable readers to quickly grasp the core content of the research, and hence contribute to analyzing hotspots and predicting development trends. The betweenness centrality calculated by CiteSpace represents the number of times a node serves as the bridge of the shortest path between two other nodes. Nodes with a betweenness centrality greater than 0.1 are key nodes (Chaomei et al., 2009), and key nodes can predict hotspots with in-depth analysis and interpretation (Ren, 2021). Setting the node type to “keyword,” the time slice to “1 year” with the rest as default values, and importing 1,012 English documents into CiteSpace, we get a knowledge map of 362 nodes (*N* = 362), 2,109 connections (*E* = 2,109) and keyword co-occurrence network with a density of 0.0323 (*D* = 0.0323) (Figure 4).

In the formula proposed by Donohue (1973):


$$T=\frac{\left[-1+\sqrt{\left(1+8I\right)}\right]}{2}$$

I is the number of keywords, and *T* is the threshold of high-frequency keywords. By calculating the threshold, high-frequency words can be quickly found (Wei and Tang, 2016). According to the information presented by CiteSpace, I value is 362, and then T ≈ 26.41 is calculated, that is, the list of high-frequency keywords with a frequency greater than 26 is obtained (Table 3). Therefore, combining the knowledge map of keyword co-occurrence (Figure 4) and the high-frequency keyword list (Table 3), screening the nodes with a frequency greater than 26 and betweenness centrality greater than 0.1, we can get key words such as “national identity,” “identity,” “attitude” and “ethnic identity.”

**Table 3:** High-frequency keywords related to ethnic cultural identity research (frequency ≥ 26).

No.	Frequency	Betweenness centrality	Year	Keywords
1	205	0.18	2012	National identity
2	159	0.12	2012	Identity
3	78	0.12	2012	Ethnic identity
4	61	0.11	2013	Attitudes
5	59	0.1	2012	Acculturation
6	58	0.08	2012	Politics
7	40	0.05	2014	Identification
8	40	0.05	2012	Culture
9	36	0.06	2012	Social identity
10	35	0.05	2013	Prejudice
11	33	0.05	2013	Multiculturalism
12	32	0.07	2012	Adolescents
13	29	0.07	2012	Discrimination
14	26	0.04	2015	Cultural identity
15	26	0.06	2013	Gender

The results show that “national identity” has the highest frequency of occurrence and the largest betweenness centrality, which appears in 205 articles, accounting for 20.3% of the totality. It occurs in the lowest number of 10 articles in 2013, while in the highest number of 31 articles in 2020. Meanwhile, “identity” ranks second in terms of frequency and centrality with a total number of 159 documents, occupying 15.7%. It has the largest number of occurrences in 23 articles in 2019 and 2022, respectively. International researchers have creatively explored ethnic cultural issues in local contexts from different perspectives. From the perspective of cross-culture network, Mao and Shen (2015) find that the changed cultural identity of expatriates is the interaction between personal choices, organization of cross-cultural relations, and individual host country background, in which individuals play a key role in shaping their own cultural identity. Employing the structural equation model, Reijerse et al. (2013) verify that the cultural representation conforming to the symbolic form of local nationalism, highly valued by multi-ethnic countries, exert prominent influence on cultural identity, which can better explain immigration attitudes and promote the harmonious coexistence of immigrants and indigenous people. Long et al. (2021) conclude that collectivists can obtain a sense of anxiety relief from collective identity when faced with difficulties that require joint efforts after studying the relationship between ethnic identity and COVID-19 anxiety. Other keywords such as “acculturation,” “politics,” “identification,” “culture” also reflect the research focus to some extent, although their betweenness centrality is not higher than 0.1. These keywords demonstrate the research hotspots from different aspects by interlinking with other related keywords, such as cultural identity of teenagers from bicultural backgrounds (Manzi et al., 2014), the relationship between cultural factors and sociopolitical status (Gidron and Hall, 2017), the relationship between positive ethnic identity and functioning of health and adaptability (Rivas-Drake et al., 2014).

#### 3.3.2. Analysis of keyword cluster

**Figure 5:**
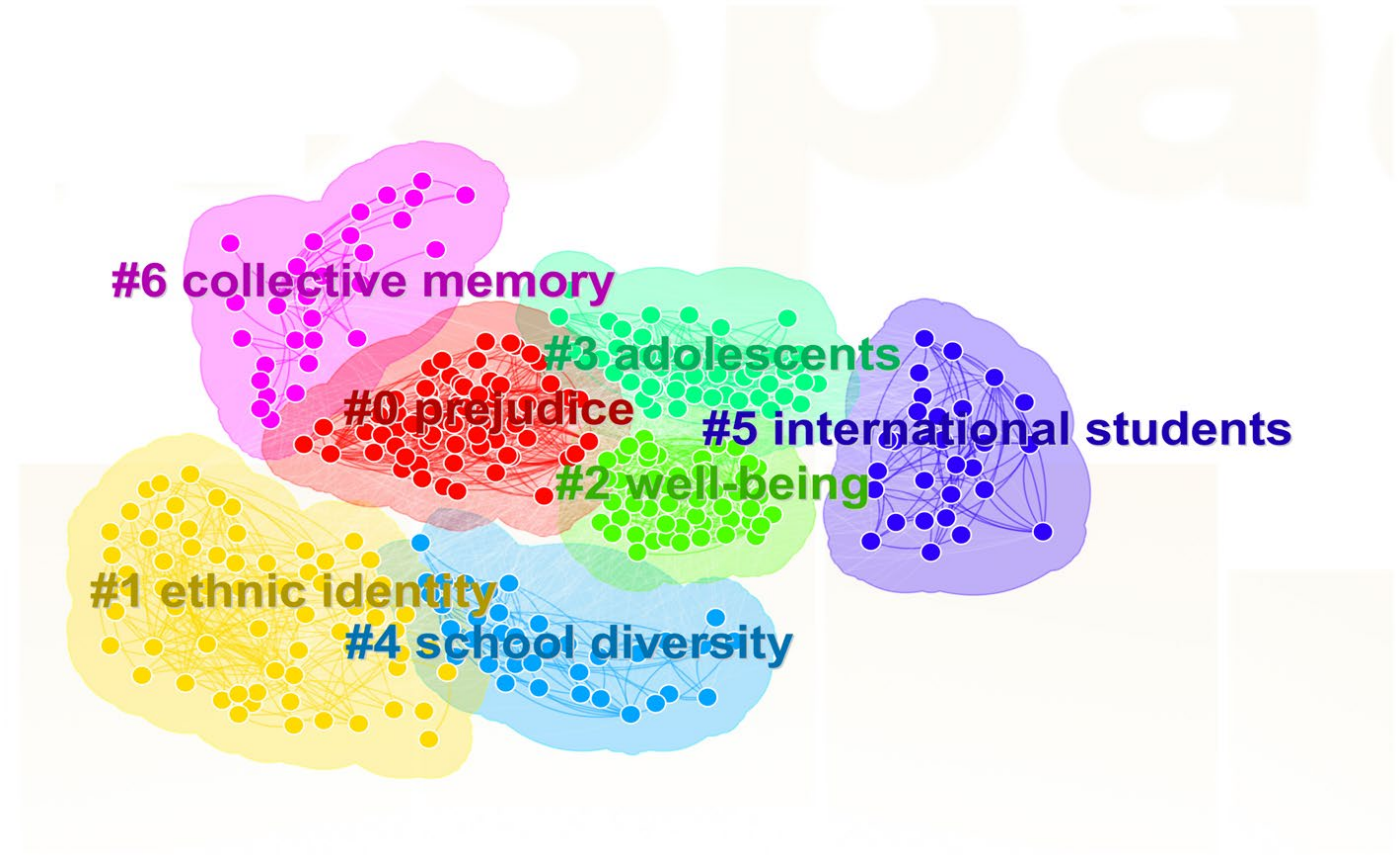
Keyword cluster map.

Keyword clustering analysis is a measurement of closeness between keywords using a similarity scale, which can serve a basis for classified statistics (Yan et al., 2022). The analysis can help researchers understand the commonality among hotspots, which is conducive To systematic analysis. Selecting “LLR” algorithm for cluster analysis on keywords, we can obtain a cluster map. As is shown in Figure 5, the module value *Q* = 0.3439, which means that The cluster structure is significant; at the same time, the average profile *S* = 0.7337, which means that the clustering result is convincing (Jie and Chaomei, 2015).

The keyword cluster map shows a total of 7 cluster categories namely #0 prejudice #1 ethnic identity #2 well-being #3 adolescent # 4 school diversity #5 international student and #6 collective memory. The original clusters can divided into three categories after manual sorting and induction as is shown in Table 4.

**Table 4:** Table of keyword cluster.

Categories of cluster	No. of cluster	Name of cluster
Emotional perception	#0, #2	Prejudice, well-being
Process of multicultural identity	#3, #4, #5	Adolescent, school diversity, international student
Cultural adaptability	#1, #6	Ethnic identity, collective memory

The first category is on emotional perception, which mainly includes #0 prejudice, and #2well-being, involving high-frequency keywords such as “multiculturalism,” “attitudes,” “discrimination,” “depression” and “adjustment.” Scholars mainly explore the relationship between national cultural identity and negative or positive emotions and psychology from the perspective of emotional perception of individuals or groups from multicultural backgrounds. Huguley et al. (2019) point out that family groups can promote the formation of a strong sense of identity by guiding young members to understand their own ethnicity, race and related cultures, which is conducive to the shaping of ethnic (racial) identities. Ethnic (racial) identity and ethnic (racial) socialization help youth get rid of the plight of racial discrimination, and the positive meaning of ethnic group membership enable them to better understand and experience the world (Neblett et al., 2012). This positive ethnic (racial) emotion plays an important role in adjusting social psychology and reducing its inherent health risks (Rivas-Drake et al., 2014), hence better guiding internal members to shape correct values. Due to variances in local conditions and customs, people may have certain differences in national cognition and cultural acceptance, both of which bring a common core emotion, that is, self-confidence (Huang et al., 2022). This self-confidence, which comes from the confidence endowed by the entire nation and country, is also the endogenous force for group members to maintain independent thinking and strengthen national identity when faced with multicultural conflicts. Generally speaking, members with a high sense of national identity and happiness are more likely to be positive and optimistic about the future development of the country (Chen et al., 2019). At the same time, due to the intensification of globalization process, the ideological level of the society has largely surpassed the cultural differences in the traditional sense, and individual members are encouraged to actively go out of the traditional setting, carry out cross-cultural communication and multiculturalism baptism, respect others’ cultural values and identities, and build a harmonious and pluralistic society in cooperation with multiple parties (Taylor-Gooby and Waite, 2014).

The second category is on the process of multicultural identity, in which #3 adolescent contains keywords such as “culture,” “marginalization” and “cultural identity,” #4 School diversity includes keywords such as “assimilation” and “care-work,” while #5 international student brings together “life course,” “expatriate adjustment” and “school adaptation.” From the keywords covered in the above clusters, it can be seen that the process of multicultural identification by international students and workers is still a hot spot for many scholars. Due to socioeconomic factors, bicultural identities frequently grabbed the public attention. The research demonstrates a positive relationship between bicultural identities, psychological adaptation, and sociocultural adaptation, hence bicultural individuals have stronger adaptability, flexibility, and a stronger sense of cultural pride and belonging (Nguyen and Benet-Martínez, 2013). Young people are the future and hope of a nation, so the research on the process of their learning and cultural adaptation from multicultural backgrounds will help build a diverse and harmonious social relationship. Umaña-Taylor et al. (2014) point out that family relations with strong ethnic ties will prompt their members to actively explore ethnic relations during adolescence, and that it is very important to place ethnic identity in other identity contexts, which is conducive to strengthening their sense of belonging to group culture. Education plays a key role in the process of strengthening multicultural identity. Local and international students should be helped to eliminate potential conflicts in terms of class, race, language, culture, etc., to provide a reasonable space for dialogue in order to better promote the practice and development of international education in the world (Jia et al., 2022). At the same time, students should also be actively encouraged to establish good international friendships, which can produce positive emotional and social support for them to reduce the intense pressure of cultural adaptation brought about by culture shock (Sheng et al., 2022). In the interweaving cultural exchanges, it is thought-provoking how ethnic minority groups can find appropriate access to their own culture and other mainstream cultures. Ozer et al. (2017) points out that ethnic cultural elements, compatible with new cultural elements, can not only protect unique national culture, but also integrate its uniqueness into multi-cultures in globalization and cross-culture exchange.

The third category is on national cultural adaptability, in which #1 ethnic identity and #6 collective memory contain keywords such as “cultural policy,” “identity politics,” “national identity,” “media” and “cultural trauma.” These keywords are all about how to realize one’s own cultural identity, how to better adapt to new life in different cultural backgrounds, and how to realize the interaction and integration of one’s own culture identity and new culture. Acculturation is a continuous and dynamic process. The dynamic dialogue between knowledge construction and identity recognition can be used as a reasonable theoretical tool to help us better understand this process. At the same time, complex acculturation involves identity changes of self and others, as well as religions, traditions and customs, which are connected with all areas of life and the social relationships other than cultures (Andreouli, 2013). Acculturation process is conditional and relative, since ethnic groups feature different cultures, histories, values and beliefs (Bornstein, 2017). Therefore, ethnic particularity and consciousness hidden in language should be taken into consideration in formulating cultural policies (Zhao and Lu, 2022). Researchers should start with common memory and perception to avoid conflicts caused by cultural system mismatch, which can hinder cultural exchanges. Cultural factors are crucial regulating factors. The high identity of foreign tourists with the culture of the host country will in turn enhance their pleasure and value perception, and stimulate their participation in cultural activities, thereby further enhancing the communication and recognition between multiculturalism (Zhang S. et al., 2019). Cultural activities are precipitated into cultural heritage, which shows the history, traditional culture and social cohesion of the host country from different aspects. Therefore, the inheritance and protection of cultural heritage will help bolster up a good international image (Liu et al., 2014). Festival celebrations are a better way to inherit traditional culture. Some methods like festival tourism can strengthen collective identity and memory, and improve experience quality and perceived value of festival activities with more innovations, ultimately enhancing the sense of belonging and identity of tourists (Zhang et al., 2021). The traditional festivals play an important role in the inheritance and dissemination of culture. Zou et al. (2021) demonstrates that that the Lantern Festival strengthens the local collective memory and identity through its strong social attributes, embodies the national culture, and promotes the inheritance of national culture. Zhang S. et al. (2019) finds that recreational festivals linked with local people’s national identity can dilute colonial culture and better shape national and ethnic identities after Macao’s return. Major cultural events often arouse citizens’ patriotic sentiments and national identity, which can be maintained through mass media to enhance their coverage and influence (Chen et al., 2019).

#### 3.3.3. Analysis of research trend

**Figure 6:**
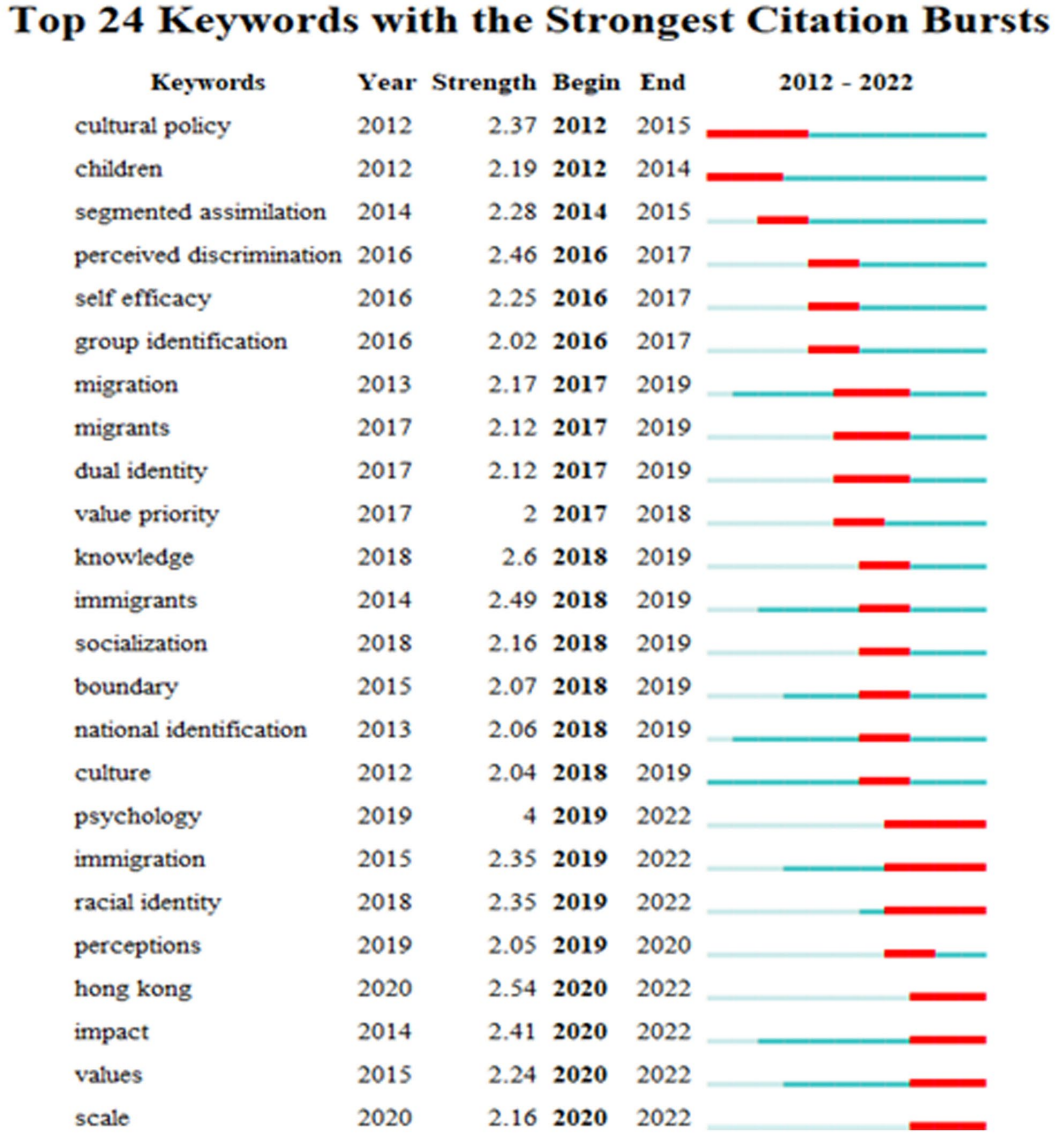
Emergent keyword map.

**Figure 7:**
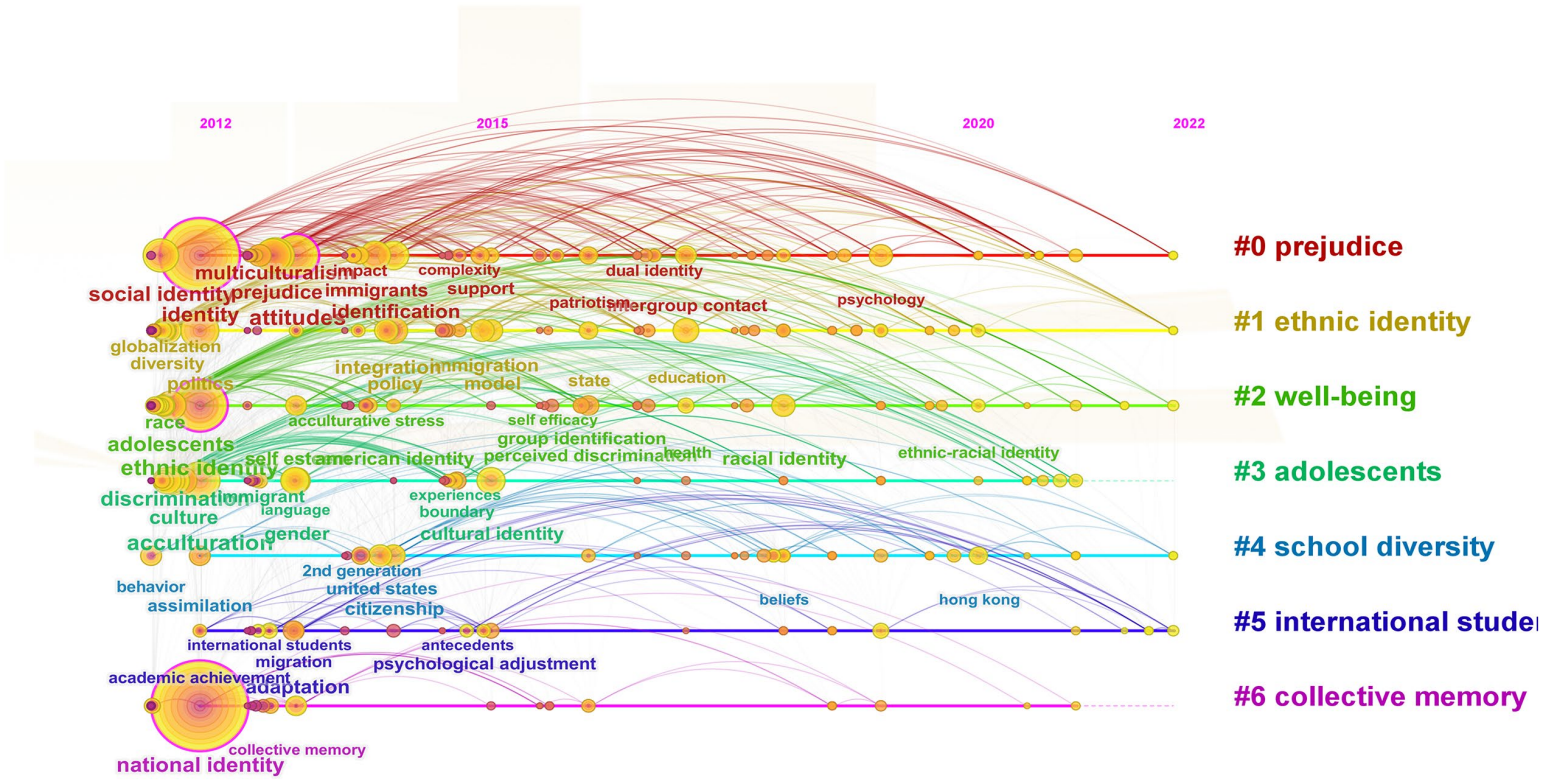
Keyword time zone map.

The emergent keywords can analyze the topics with greater influence over a period of time, and show the span between the first appearance and the end of the keyword, which can help researchers better analyze the development trend (Wang and Sun, 2020). The keyword time zone map can vividly reflect the evolution of hotspots along with the timeline, which is helpful to accurately grasp the development of hotspots. Based on the existing literature, the emergent keyword map (Figure 6) and the keyword time zone map (Figure 7) are drawn.

Through the comprehensive analysis of the two graphs, we can find that the research evolution path can be divided into three stages. The first stage is from 2012 to 2016, when the research develops stably. This stage includes 6 prominent words, among which “perceived discrimination,” “cultural policy” and “segmented assimilation” rank top 3 in terms of intensity. At this stage, researchers focus on the interaction between original and dominant cultures (Samnani et al., 2013), risks and opportunities brought about by diversified development (Jensen and Arnett, 2012), cultural conflicts between individuals and multi-culture groups (Martiny et al., 2012), and identity integration effects (Swann et al., 2012). Meanwhile, researchers also pay attention to the identity and autonomy issues that easily trouble adolescent growth (Fuligni and Tsai, 2015), and the stronger resilience of members of bicultural identity to adversity and discrimination (Huynh et al., 2014) and adaptability (Sirikantraporn, 2013).

The second stage is from 2017 to 2019, when the research grows rapidly. Although this stage is not long, there are many words that emerge, with a total of 14 prominent words. Among them, psychology has the strongest degree of emergence, which shows that many scholars tend to link national cultural identity to psychology to build interdisciplinary disciplines since psychological approach to the search is significant. Researchers are mainly interested in adaptation of immigrants and groups with dual identity backgrounds to society (Ferguson et al., 2017) and their psychological and behavioral impacts and changes. On the one hand, complex bicultural identity will lead individuals to produce positive psychological and sociocultural outcomes. On the other hand, it will lead to potential risks in intergroup relations (Chu et al., 2017). It is of positive significance for youngsters to explore how to fully display their subjective initiative and consciously adjust their cultural identities in interactive behaviors to so that they can adapt to the social environment of globalization in the new era (Ferguson et al., 2017). Cultural identity is a dynamic process rather than a static one. Ethnic policies vary with social backgrounds and national conditions with the aim to eliminate prejudice and discrimination (Luo, 2019), strengthen social bonds, resolve cultural conflicts and build cultural intimacy (Kania-Lundholm and Lindgren, 2017), reconstruct local social entities (Sun et al., 2019).

The third stage is from 2020 to 2022, when ethnic cultural identity grows steadily. There are four emergent keywords at this stage, namely Hong Kong, impact, value, and scale. Researchers revolve around keywords such as national pride, trust, cultural intelligence, association, stress, ethnic-racial identity, host country nationals and depression. They focus on strengthening national identity, shaping the form and means of cultural identity and its dual impact (Zhang et al., 2021; Chow-Garcia et al., 2022). Cross-cultural psychologists point out that through the intermediary role of cultural identity, an individual’s psychological state or identity perception will change, leading to corresponding behavioral changes (Zhang et al., 2021). The impact of cultural identity is multifaceted including national identity (Zhang et al., 2021), identity confusion (Cheon et al., 2020), sense of belonging (Chow-Garcia et al., 2022), network socialization (Cai et al., 2022), cultural plasticity (Gagnon, 2022), mental health (Li et al., 2022), and psychological adjustment of adolescents (Safa et al., 2022).

## 4. Conclusion and discussion

From the WOS database, a visual analysis of the literature on cultural identity in the past decades was conducted, based on bibliometrics and knowledge graph research methods. The results show that cultural identity has become a topic of common concern to many international scholars, whose research covers the disciplines including psychology, sociology, ethnology, education and political science, with focus on cultural issues such as ethnic conflicts, immigrant groups, cultural identity process, cultural adaptability and national unity. On the one hand, the research on national cultural identity has distinctive characteristics of regions, nationalities and nations. On the other hand, the joint efforts between different countries and regions are emphasized to actively build mutually-understanding and harmonious community with a shared future for mankind while focusing on national identity and cultural identity. At the same time, a quantitative analysis of researchers shows inadequate international cooperation among research institutions in this field with a high degree of dispersion, which indicates that cross-cultural international research group has not been formed. Global cooperation among cross-cultural research institutions needs to be strengthened to achieve the ultimate goal of building a community with a shared future for mankind in a multicultural context. In addition, “national identity” is the keyword with the highest frequency of occurrence and the largest betweenness centrality, indicating the research of national identity is still the core issue that researchers are most concerned about. It also reflects the roots of extreme occurrences of nationalism in certain countries and regions, hence more emphasis should be put on cultural identity between countries, rather than narrow national identities.

In fact, complex cultural identity not only covers different countries, ethnic groups, regions and even social groups, but also involves individual cognition, emotion and cultural adaptability. It is important for formulating national policies, developing cultural and educational undertakings, or solving the contradictions and conflicts of globalization. Therefore, in order to properly solve this series of practical problems, scholars should work together and collaborate to strengthen cultural interaction and exchanges between ethnic groups and countries, so as to actively promote and construct a harmonious community with a shared future for mankind.

Limitations of this study: (1) The results of the analysis mainly rely on the downloaded journal documents and the knowledge map drawn on this basis, while the research results on ethnic cultural identity are of various types, including conference reports, dissertations, monographs. This study has not incorporated other content into the statistical source, so the results of the analysis have certain limitations. (2) We do not grasp the comprehensive international situation and conduct an in-depth analysis of some factors affecting national cultural identity in other countries, so the research results are not all-embracing. (3) This study only uses CiteSpace software to draw the knowledge map, and the results inevitably have some deviations. In subsequent studies, multiple software can be used in order to obtain more comprehensive and accurate analysis.

## Author contributions

LK: Conceptualization, Data curation, Methodology, Visualization, Writing – original draft. XG: Writing – review & editing. BL: Supervision, Visualization, Writing – review & editing. JW: Funding acquisition, Supervision, Writing – review & editing.
